# Leadless Pacemaker for Sinus Pauses and Syncope in Pregnancy: A Feasible Alternative to Conventional Pacing

**DOI:** 10.19102/icrm.2026.17032

**Published:** 2026-03-15

**Authors:** Miki Yokokawa, Shaurya Srivasta, Ali H. Sheikh

**Affiliations:** 1 Division of Cardiology, University of Michigan Health Sparrow Health System, Lansing, MI, USA; 2 Division of Cardiac Electrophysiology, University of Michigan Health Sparrow Health System, Lansing, MI, USA

**Keywords:** Fluoroscopy, leadless pacemaker, pregnancy, sinus pause, syncope

## Abstract

Bradyarrhythmias with syncope during pregnancy present a complex and multifaceted challenge for both patients and health care providers. When device implantation is indicated for bradyarrhythmia-associated syncope during pregnancy, the procedure should be performed with particular attention to minimizing fluoroscopy exposure to ensure maternal and fetal safety. A 29-year-old woman at 15 weeks of gestation with sinus pauses presented with recurrent syncopal episodes. The patient underwent Micra™ AV leadless pacemaker implantation (Medtronic, Minneapolis, MN, USA) with a radiation exposure of 26.5 mGy and a total fluoroscopy time of 2 min, which is generally considered a negligible dose during pregnancy, based on the recommendations of the National Council on Radiation Protection and Measurements. In pregnant patients, a leadless pacemaker may offer a viable alternative to conventional pacing, primarily due to its reduced radiation exposure, shorter procedure time, minimally invasive approach, absence of transvenous leads, and lower risk of site infection.

## Introduction

Pregnant patients with irreversible symptomatic bradycardia associated with syncope may be at an increased risk of adverse maternal and/or fetal outcomes. In such cases, permanent pacemaker implantation is recommended, with particular attention to minimizing radiation exposure.^1^ The newer technologies allow for performing device implants with minimal or even zero fluoroscopy.^2–4^ We report a case of a patient at 15 weeks of pregnancy who underwent leadless pacemaker implantation with minimal radiation exposure.

## Case presentation

A 29-year-old woman, G3P1011 at 15 weeks of gestation, with a history of palpitations and prior loop recorder implantation presented with recurrent syncopal episodes. Her cardiac history included orthodromic atrioventricular reciprocating tachycardia, previously treated with radiofrequency ablation of a right posterior septal accessory pathway at an outside facility. The procedure was complicated by a posterior wall myocardial infarction due to occlusion of the posterolateral branch of the right coronary artery, requiring emergent drug-eluting stent placement. She subsequently underwent an additional radiofrequency ablation for a mid-septal accessory pathway. She then experienced recurrent symptoms and underwent two electrophysiology studies, both of which were non-inducible. There was no evidence of atrioventricular nodal conduction abnormalities or sinus node disease. A loop recorder was implanted for ongoing monitoring.

**Figure 1:**
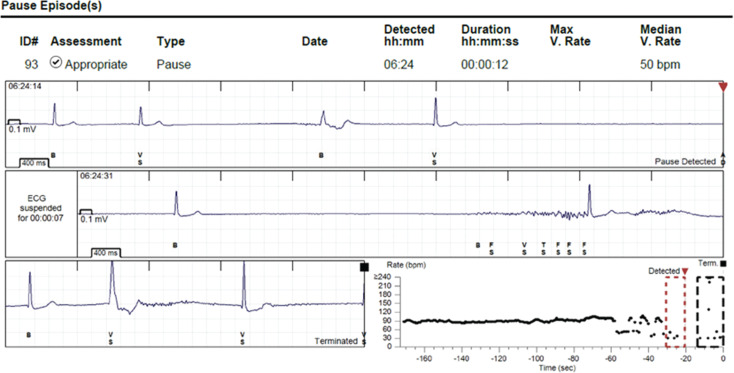

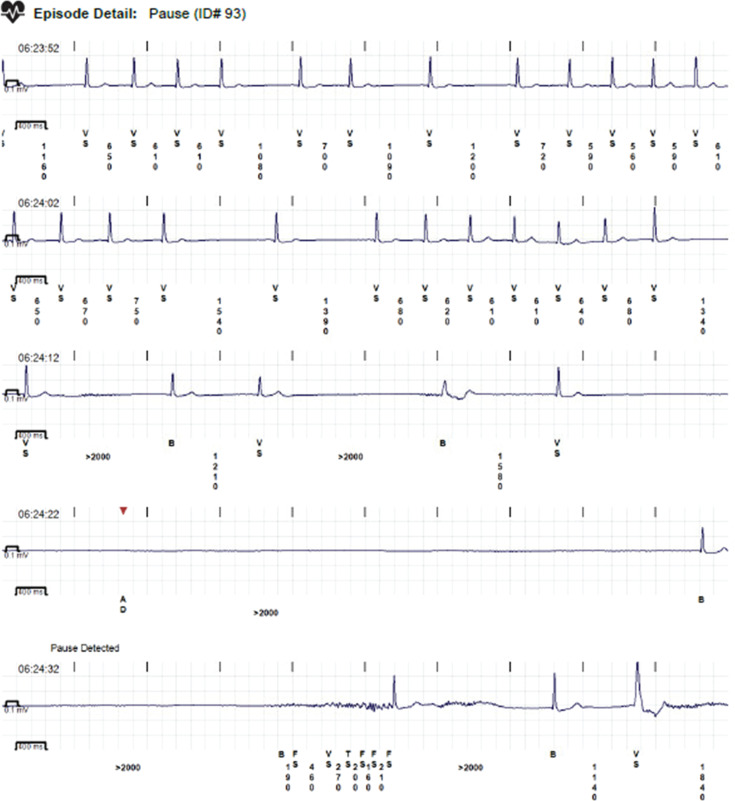
Loop recorder interrogation showing a 12-s sinus arrest associated with syncope.

During her second pregnancy a few years earlier, the patient experienced hyperemesis gravidarum and was found to have vagally mediated sinus pauses lasting up to 10 s. A cesarean section was performed to avoid the Valsalva effort associated with vaginal delivery. During her third pregnancy, she developed recurrent syncopal episodes with sinus pauses; later episodes of sinus arrest occurred without any clear precipitating events. An echocardiogram demonstrated a normal ejection fraction with no wall motion abnormalities. A 12-lead electrocardiogram showed no evidence of pre-excitation. Loop recorder interrogation revealed episodes of atrial tachycardia followed by sinus arrest lasting up to 16 s **(Figure 1)**. Given the malignant nature of the patient’s syncope, definitive treatment with pacing support was deemed necessary to prevent both maternal and fetal harm. A comprehensive multidisciplinary team discussion was conducted, involving electrophysiology, maternal–fetal medicine, and anesthesiology to ensure optimal outcomes for both mother and fetus. A thorough assessment was performed, including evaluation of maternal cardiac status, comorbidities, and obstetric risk factors. A shared decision-making process was undertaken after comprehensive counseling on the potential fetal risks associated with sedation and fluoroscopic exposure, as well as the benefits, alternatives, and long-term device implications. The Micra™ AV leadless pacemaker (Medtronic, Minneapolis, MN, USA) was considered the most suitable option, as it minimizes radiation exposure to both the patient and the fetus during fluoroscopy. Informed consent was obtained, and the patient agreed to proceed with pacemaker implantation.

**Figure 2:**
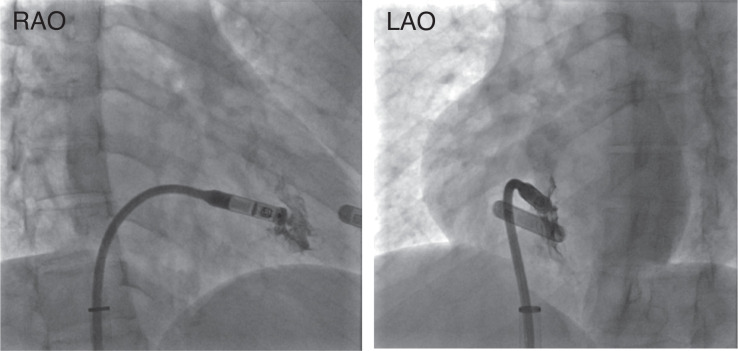
Multiplanar fluoroscopy with a right ventriculogram, which was used to confirm the proper placement of the leadless pacemaker device. *Abbreviations:* LAO, left anterior oblique; RAO, right anterior oblique.

**Figure 3:**
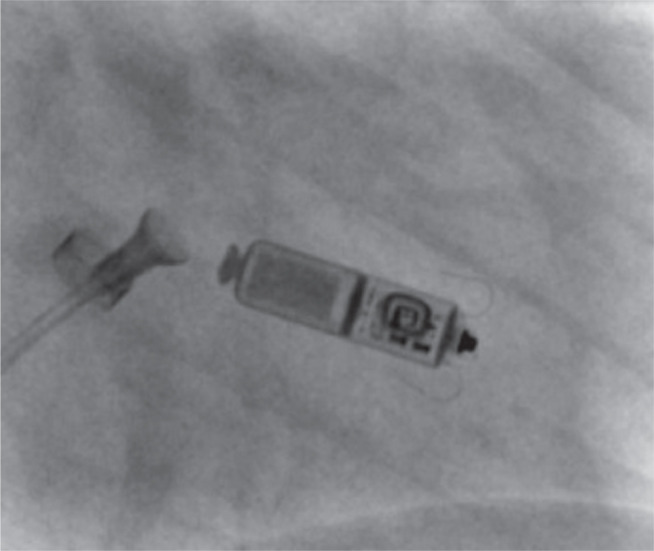
Tug test under direct fluoroscopy. After confirming adequate pacing parameters, a tug test was performed under direct fluoroscopy to verify secure device engagement.

**Figure 4:**
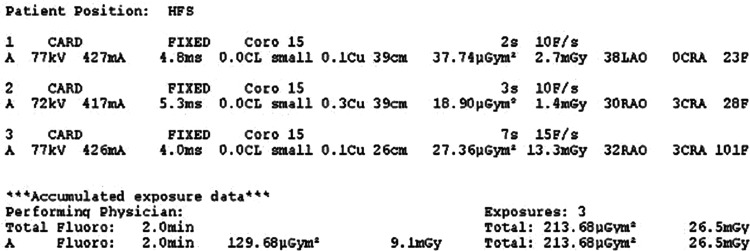
Fluoroscopy parameters. The total fluoroscopy time was 2 min, with a radiation dose of 26.5 mGy.

The patient was brought to the electrophysiology laboratory and prepped and draped in a sterile manner. Standard radiation precautions were followed, and additional protective measures were implemented by shielding the gravid uterus with a lead apron (≥0.25 mm Pb) to further minimize fetal radiation exposure. All anesthesia care was provided by a certified registered nurse anesthetist under the supervision of an anesthesiologist. The patient received intermittent doses of fentanyl, and local anesthesia was achieved with lidocaine administered to the skin overlying the right groin. Using the Seldinger technique under direct ultrasound guidance, the right femoral vein was accessed. Following the insertion of a short 8-Fr sheath, an Amplatz Super Stiff 180 cm, 0.035″ guidewire (Boston Scientific, Marlborough, MA, USA) was advanced into the superior vena cava, and the short sheath was subsequently removed. Sequential femoral vein dilation was performed using 8-, 12-, 16-, 20-, and 24-Fr introducers, with careful blunt dissection around the venotomy site to facilitate smooth passage. Once the final dilation was completed, the introducer system was advanced over the guidewire under direct fluoroscopic guidance into the right atrium. With the access sheath positioned in the right atrium, the pacemaker introducer system was carefully advanced, and the introducer sheath was withdrawn into the inferior vena cava. Upon reaching the right ventricle, the introducer system was rotated to achieve optimal positioning at the septal wall. Multiplanar fluoroscopy and a right ventriculogram using 15 mL of a 50:50 mixture of contrast and saline were performed to confirm appropriate placement at the mid-to-high right ventricular septum **(Figure 2)**. Collimation and minimized magnification were employed to restrict radiation exposure to the region of interest. Once the position was verified, the device was successfully deployed. After confirming acceptable pacing parameters, a tug test was performed under direct fluoroscopic visualization, confirming engagement on at least two occasions **(Figure 3)**. With stable pacing parameters and a successful tug test, the suture port was flushed, and the suture was cut and removed under fluoroscopic guidance. The total fluoroscopy time was 2 min, with a total radiation dose of 26.5 mGy **(Figure 4)**. The impedance at implantation was 720 Ω, with intrinsic R-waves measuring 6.8 mV. The pacing threshold was 0.25 V at 0.4 ms, and the device was programmed in the VDD mode at a lower rate limit of 50 bpm. Close maternal and fetal monitoring was performed after the procedure. The patient had an uneventful recovery and was discharged without complications. Device management during labor and delivery was coordinated with input from all relevant specialties. At the routine 6-month follow-up after implantation, the patient remained asymptomatic, with a pacing burden of <1.0%.

## Discussion

The prevalence of syncope during pregnancy has been reported to range from 0.3%–9.0%.^5^ Physiological changes in pregnancy, including increased blood volume, decreased systemic vascular resistance due to vasodilation, and hormonal fluctuations, can disrupt autonomic regulation and increase susceptibility to syncope. Additionally, the growing uterus may compress the vena cava, reducing venous return and cardiac output, which can precipitate syncope, particularly when the patient is in the supine position during late pregnancy.^6^ Prenatal maternal syncope is an independent risk factor for intrauterine growth restriction, cesarean delivery, and long-term neurological morbidity in the offspring.^7,8^ A comprehensive understanding of the incidence, diagnosis, and management of syncope in pregnancy is essential to ensure optimal maternal and fetal outcomes. Early recognition and appropriate management of cardiac syncope are critical, as it is associated with an increased risk of adverse maternal and neonatal outcomes. Cardioneuroablation is an emerging catheter-based therapy for cardioinhibitory syncope; however, its safety and role during pregnancy have not been established.^9^ In this case, given the malignant nature of the patient’s syncope, definitive treatment with pacing support was indicated to prevent maternal and fetal harm.

When pacemaker implantation is indicated during pregnancy, strategies to minimize fluoroscopy exposure should be implemented due to concerns regarding potential fetal radiation risks. Studies have shown that the risk of radiation-induced adverse events, such as fetal defects or miscarriage, increases proportionally with gestational age up to 20 weeks.^10^ According to the Heart Rhythm Society expert consensus statement on the management of arrhythmias during pregnancy, the fetal radiation dose from most common cardiovascular interventions is unlikely to exceed the 50 mGy threshold, which is considered a negligible risk for excess malignancy.^1^ Although fluoroscopy times vary with operator technique and institutional protocols, the average duration for standard single- or dual-chamber pacemaker implantation has been reported to be 5–8 min, corresponding to an estimated radiation dose of approximately 20–110 mGy.^11^ Lower fluoroscopy times can be achieved with three-dimensional navigation techniques. Three-dimensional electroanatomic mapping systems are increasingly used for fluoroscopy-free permanent pacemaker implantation^3,4^; however, these procedures are technically demanding and often require longer procedure times.^12^ Shah et al. reported a case of leadless pacemaker implantation (Micra™; Medtronic) in a 31-week pregnant patient with intermittent complete heart block and recurrent syncope, with radiation exposure maintained at <40 mGy.^2^

A previous study demonstrated that complications associated with leadless pacemaker implantation are predominantly intraprocedural.^13^ Patient selection and anatomical considerations are crucial for optimal outcomes with leadless pacemaker implantation. In difficult anatomical scenarios, the leadless approach may require multiple deployment attempts and increased manipulation, which can further increase fluoroscopy time and procedure duration. The advantages of leadless pacemakers stem mainly from the elimination of lead-related complications such as pneumothorax, dislodgement, fracture, insulation failure, and pocket-related complications, including hematoma, erosion, and infection, which together account for >70% of complications in transvenous pacemaker implantation.^13,14^ In the general population, large multicenter studies show that leadless pacemakers carry lower rates of device-related complications (0.5%–1.8% at 1 year) compared to transvenous pacemakers (1.9%–7.4%), with most transvenous complications being lead- or pocket-related.^13^ Leadless systems also have a lower risk of device infection (<0.2% vs. 0.7%) and reintervention (38%–41% lower in the first year).^15^ However, there are no comparative data to guide device selection in pregnant patients.

The use of leadless pacemakers should also be guided by factors such as the anticipated frequency of right ventricular pacing and baseline ventricular function to optimize implantation strategies and refine patient selection. Single-chamber ventricular leadless pacemakers do not support atrial pacing or consistent atrioventricular synchrony. Patients with intact sinus function may be suitable candidates for single-chamber ventricular leadless pacing when pacing needs are infrequent, such as in cases of rare but clinically significant sinus pauses.^16^ In patients with vasovagal syncope, the purpose of pacing is to prevent syncope precipitated by abrupt bradycardia or asystole rather than to address underlying heart rate abnormalities,^17^ and these patients generally exhibit a low pacing burden. One study reported a pacing percentage of 1% ± 1% in patients with cardioinhibitory vasovagal syncope compared with that of 37% ± 43% in other pacing indications.^18^ Another study demonstrated that the safety and efficacy of single-chamber leadless pacemakers were comparable to those of conventional dual-chamber transvenous systems in patients with cardioinhibitory vasovagal syncope on intermediate-term follow-up.^19^ Taken together, these findings suggest that leadless pacemaker implantation may be a reasonable option in patients with prolonged asystole due to cardioinhibitory vasovagal syncope. The recent development of dual-chamber leadless pacing systems has expanded the clinical indications for leadless pacemaker therapy.^20^

Device longevity and the potential need for future replacements remain critical considerations in younger patients. In patients with vasovagal syncope during pregnancy, pacemaker implantation should be reserved for cases of irreversible symptomatic bradycardia that pose risk to maternal or fetal health. Routine device reimplantation is not indicated unless there is a clear, guideline-based need.^21^ Leadless pacemakers generally offer a longer battery life than transvenous devices; however, extraction after prolonged dwell times can be more complex, particularly when the device becomes encapsulated by fibrosis.^14,22^

Intravenous contrast is more frequently required in Micra™ leadless pacemaker implantation than during the implantation of transvenous systems to confirm positioning and deployment. Its use should be restricted to situations of clear procedural necessity, balancing maternal benefit against potential fetal risk. Although iodinated contrast crosses the placenta and may theoretically affect fetal thyroid function, available human data have not demonstrated teratogenic effects, an increased risk of miscarriage, or clinically significant neonatal thyroid dysfunction.^23,24^ However, when contrast administration is essential for safe implantation, its use is justified but should be minimized, and postnatal thyroid monitoring is recommended following substantial exposure.

In summary, there are no robust data directly comparing maternal or fetal outcomes between Micra™ leadless and transvenous pacemaker implantation during pregnancy. Clinical recommendations emphasize a multidisciplinary approach involving maternal–fetal medicine, cardiology, and anesthesiology, with comprehensive counseling, documentation, and individualized risk–benefit assessments, along with advance planning for potential complications during labor and delivery.

## Conclusion

In patients with cardioinhibitory vasovagal syncope during pregnancy, minimizing fluoroscopy exposure is crucial to ensure maternal and fetal safety. Micra™ leadless pacemakers may represent a viable alternative to conventional systems, offering reduced radiation exposure and avoidance of lead- and pocket-related complications, features that are particularly advantageous during pregnancy. However, their limitations in pacing options must be carefully weighed during patient selection.
